# COPD Assessment Test Changes from Baseline Correlate with COPD Exacerbations: A Longitudinal Analysis of the DACCORD Observational Study

**DOI:** 10.1007/s00408-020-00357-y

**Published:** 2020-05-04

**Authors:** Peter Kardos, Claus F. Vogelmeier, Heinrich Worth, Roland Buhl, Victoria Obermoser, Carl-Peter Criée

**Affiliations:** 1 Group Practice and Centre for Allergy, Respiratory and Sleep Medicine, Red Cross Maingau Hospital, Praxis: Friedberger Anlage 31-32, 60316 Frankfurt, Germany; 2 Department of Medicine, Pulmonary and Critical Care Medicine, University Medical Centre Giessen and Marburg, Philipps-University Marburg, Member of the German Centre for Lung Research (DZL), 35043 Marburg, Germany; 3 Facharztforum Fürth, 90762 Fürth, Germany; 4 Pulmonary Department, Mainz University Hospital, 55131 Mainz, Germany; 5 Clinical Research, Respiratory, Novartis Pharma GmbH, 90429 Nürnberg, Germany; 6 Department of Sleep and Respiratory Medicine, Evangelical Hospital Goettingen-Weende, 37120 Bovenden, Germany

**Keywords:** Acute exacerbations of COPD, Bronchodilator, Chronic obstructive pulmonary disease, COPD course and therapy, Health-related quality of life

## Abstract

**Purpose:**

A number of analyses have shown the immediate impact of COPD exacerbations on health status. However, none evaluated the long-term correlation between health status and the occurrence of exacerbations.

**Methods:**

DACCORD is an observational study in patients with COPD recruited across Germany following initiation or change in COPD maintenance medication. Data collected include COPD Assessment Test (CAT) total score on entry and after 1 and 2 years, and the occurrence of exacerbations. We analysed the correlation between change from baseline in CAT total score and exacerbations, after excluding patients who exacerbated during the quarter immediately prior to the CAT assessment of interest.

**Results:**

The initial correlation analysis was performed in 6075 patients, 28% with ≥ 1 exacerbation over the 2-year follow-up, and 58% with a clinically relevant CAT improvement. There was a significant correlation between exacerbations over 2 years and CAT change from baseline at Year 2 (*p* = 0.0041). The Spearman’s correlation coefficient was 0.03711, indicating very weak correlation—potentially driven by the high proportion of non-exacerbating patients. In a subsequent logistic regression, the probability of experiencing frequent (≥ 2 per year) or severe exacerbations was higher in patients with worsening in CAT total score (*p* < 0.001). However, the probability of a patient exacerbating in Year 1 or Year 2 did not correlate with CAT change.

**Conclusions:**

In this population (initiating or changing maintenance COPD medication), patients with frequent or severe exacerbations had a long-term worsening in health status (beyond the acute effect of an exacerbation) compared with patients who do not exacerbate.

## Background

One factor potentially driving chronic obstructive pulmonary disease (COPD) progression is exacerbations, especially frequent and/or severe events [1, 2]. In one study, health status worsened at the time of the exacerbation, subsequently returning to baseline after approximately 10 days [3]. However, the recovery in health status could take as long as 12 weeks, with some patients not fully recovering, especially if they experience a second exacerbation [4]. Health status also has a predictive ability for future exacerbations, with patients who frequently exacerbate having higher (worse) baseline COPD Assessment Test (CAT) total scores [3], and higher baseline CAT associated with shorter time to first exacerbation and a higher exacerbation risk [5]. However, none of these analyses evaluated the long-term correlation between change in health status during the stable (non-exacerbation) state and the occurrence of exacerbations.

DACCORD is an observational study being conducted in primary and secondary care across Germany. We decided to analyse whether there was a correlation between change from baseline in CAT total score and the occurrence of exacerbations in DACCORD. To exclude the acute effect of exacerbations on CAT total score, we excluded patients who exacerbated during the quarter immediately prior to the CAT assessment.

## Methods

### Trial Design

As DACCORD is non-interventional, specific visits are not mandated by the protocol. However, consistent with usual care in Germany, it was anticipated that data would be recorded approximately every three months. At the baseline visit, data collected in Internet-based electronic case report forms included: demographic and disease characteristics; COPD medication; CAT; forced expiratory volume in 1 s (FEV_1_); and exacerbations in the six months prior to entry (requiring oral corticosteroids and/or antibiotics or hospitalisation). We collected six-month historical exacerbations results to minimise the potential impact of patient recall on data accuracy. Exacerbations data were then collected at three-monthly visits, with CAT data collected at the 1- and 2-year visits. Full details of the methods have been published [6], as have baseline characteristics [7], and 1- and 2-year follow-up data [8–11].

### Participants

The main inclusion criteria are a diagnosis of COPD fulfilling the German COPD Disease Management Program (DMP) criteria (one of which is that the COPD diagnosis is confirmed by spirometry), age ≥ 40 years, and initiating or changing COPD maintenance medication (between or within therapeutic class). Given the non-interventional nature, the decision to initiate or change medication had to be made by patients’ physicians prior to inclusion in DACCORD. To recruit as broad a population as possible, patients were excluded only if they were in the asthma DMP, or if they were participating in a randomised clinical trial. The study is registered in the European Network of Centres for Pharmacoepidemiology and Pharmacovigilance (EUPAS4207), and was approved by the ethics committee of the University of Erlangen-Nuremberg. All patients provided written informed consent prior to inclusion.

### Sample Size and Statistical Methods

The analyses in this manuscript include patients who completed visits at the end of Years 1 and 2, and who missed no more than one of the intermediate quarterly visits each year. To evaluate the correlation between exacerbations and CAT total score change from baseline at the end of Year 2, we excluded patients who exacerbated in the last three months of the second year of follow-up (between Visits 7 and 8), to avoid any short-term impact of exacerbations on CAT total score. The correlation was analysed using Spearman’s correlation coefficient. This population was also used to evaluate the impact of frequent or severe exacerbations on CAT total score, defined as ≥ 2 exacerbations or ≥ 1 exacerbation resulting in hospitalisation in each year of follow-up. Subgroup comparisons of CAT total score were performed using the Wilcoxon signed rank test for mean score, and the χ^2^ test for the percentage of patients with a clinically relevant (≥ 2 unit) change from baseline—either improvement or worsening.

The relationship between CAT progression (using data captured at baseline and the end of Years 1 and 2) and the occurrence of exacerbations was then analysed in two subgroups:

- Patients with a progressive worsening in CAT total score, defined as a clinically relevant worsening (≥ 2 units) from baseline at the end of Year 1 *and* a further clinically relevant worsening (a further ≥ 2 units) between the end of Year 1 and the end of Year 2.
- Patients with progressive improvement in CAT total score, defined as clinically relevant improvements from baseline at Year 1 *and* between Year 1 and Year 2.

For these analyses, in addition to excluding patients who reported an exacerbation between Visits 7 and 8, we also excluded patients who reported an exacerbation between Visits 3 and 4 (the three months prior to each CAT evaluation). The impact of frequent or severe exacerbations on CAT change in each year, and of a progressive improvement in CAT on the occurrence of exacerbations in each year were evaluated using logistic regression, including the baseline factors sex, age, smoking status, duration since diagnosis, FEV_1_% predicted, and CAT total score, and the number of exacerbations in the six months prior to entry.

## Results

### Participants

**Fig. 1:**
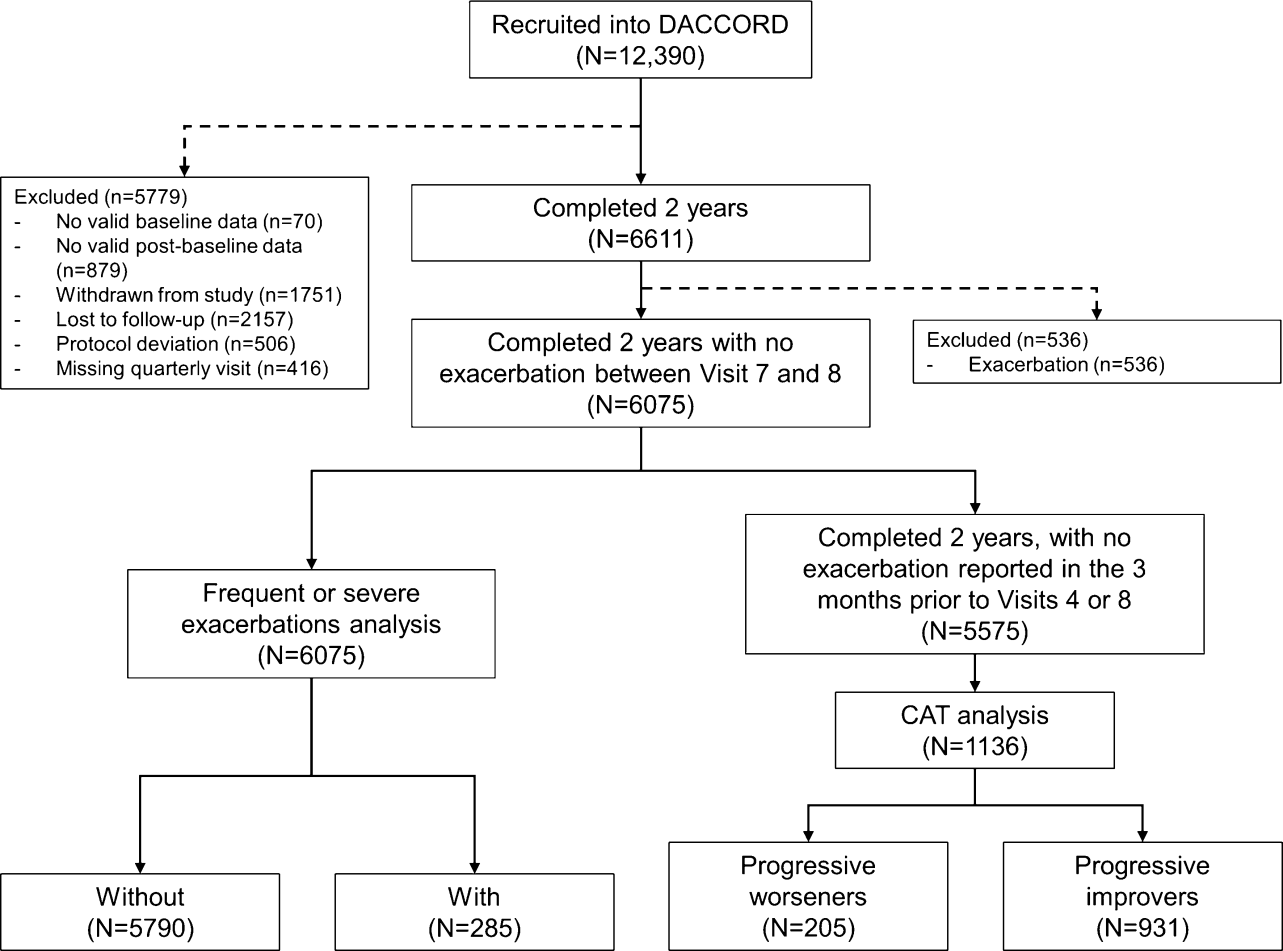
Patient disposition

Overall, 53% of patients recruited into DACCORD completed the 2-year follow-up, 6075 (92%) of whom had no exacerbation between Visits 7 and 8, and so were included in the initial analyses (Fig. 1). The majority were male, and had been diagnosed with COPD more than a year prior to entry, with just over half having moderate airflow limitation (Table 1). Exacerbations in the 6 months prior to entry were rare.

**Table 1:** Baseline demographics and disease characteristics

	Overall population (*N* = 6075)	Frequent or severe exacerbations analysis			CAT analysis		
		Without (*N* = 5790)	With (*N* = 285)	*p* value	Progressive worseners (*N* = 205)	Progressive improvers (*N* = 931)	*p* value
Sex, male, *n* (%)	3561 (58.6)	3384 (58.4)	177 (62.1)	0.242^a^	111 (54.1)	520 (55.9)	0.698^a^
Age (years), mean (SD)	66.3 (10.2)	66.3 (10.2)	66.7 (9.5)	0.448^b^	66.8 (9.7)	66.6 (10.1)	0.789^b^
Smoking status, *n* (%)				< 0.001*^a^			< 0.001*^a^
Ex-smoker	2513 (41.4)	2365 (40.8)	148 (51.9)		103 (50.2)	371 (39.8)	
Current smoker	2320 (38.2)	2218 (38.3)	102 (35.8)		74 (36.1)	328 (35.2)	
Never smoker	1221 (20.1)	1188 (20.5)	33 (11.6)		26 (12.7)	232 (24.9)	
Duration since primary diagnosis, n (%)				< 0.001*^a^			0.346^a^
≤ 1 year	1547 (25.5)	1500 (25.9)	47 (16.5)		52 (25.4)	268 (28.8)	
> 1 year	4528 (74.5)	4290 (74.1)	238 (83.5)		153 (74.6)	663 (71.2)	
FEV_1_% predicted, mean (SD)^‡^	61.6 (20.3)	62.3 (20.2)	47.3 (17.0)	< 0.001*^b^	56.1 (18.0)	64.0 (20.6)	< 0.001*^b^
FEV_1_% predicted, *n* (%)^‡^				< 0.001*^a^			< 0.001*^a^
≥ 80%	1074 (17.7)	1060 (18.3)	14 (4.9)		22 (10.7)	204 (21.9)	
50 to < 80%	3175 (52.3)	3080 (53.2)	95 (33.3)		100 (48.8)	495 (53.2)	
30 to < 50%	1569 (25.8)	1430 (24.7)	139 (48.8)		70 (34.1)	193 (20.7)	
< 30%	257 (4.2)	220 (3.8)	37 (13.0)		13 (6.3)	39 (4.2)	
CAT, mean (SD)	19.2 (7.7)	19.1 (7.7)	21.8 (7.9)	< 0.001*^b^	13.4 (6.3)	23.1 (6.6)	< 0.001*^b^
CAT score, *n* (%)				< 0.001*^a^			< 0.001*^a^
0– < 10	694 (11.4)	678 (11.7)	16 (5.6)		66 (32.2)	10 (1.1)	
10–20	2673 (44.0)	2565 (44.3)	108 (37.9)		110 (53.7)	320 (34.4)	
> 20–30	2275 (37.4)	2155 (37.2)	120 (42.1)		29 (14.1)	479 (51.5)	
> 30	433 (7.1)	392 (6.8)	41 (14.4)		0	122 (13.1)	
Number of exacerbations in the 6 months prior to entry, *n* (%)				< 0.001*^a^			0.001*^a^
0	4672 (76.9)	4506 (77.8)	166 (58.2)		175 (85.4)	674 (72.4)	
1	982 (16.2)	903 (15.6)	79 (27.7)		27 (13.2)	162 (17.4)	
≥ 2	372 (6.1)	335 (5.8)	37 (13.0)		3 (1.5)	79 (8.5)	
Missing	49 (0.8)	46 (0.8)	3 (1.1)		0	16 (1.7)	
Comorbidities, n (%)							
Alpha-1 antitrypsin deficiency	11 (0.2)	10 (0.2)	1 (0.4)	0.411^a^	1 (0.5)	1 (0.1)	0.328^a^
Bronchiectasis	65 (1.1)	57 (1.0)	8 (2.8)	0.010*^a^	1 (0.5)	12 (1.3)	0.483^a^
Bronchial carcinoma	79 (1.3)	75 (1.3)	4 (1.4)	0.787^a^	2 (1.0)	12 (1.3)	1.000^a^
Cardiovascular disease	3432 (56.5)	3262 (56.3)	170 (59.6)	0.298^a^	120 (58.5)	545 (58.5)	1.000^a^
Diabetes mellitus, type 2	1058 (17.4)	1010 (17.4)	48 (16.8)	0.873^a^	34 (16.6)	178 (19.1)	0.430^a^
Osteoporosis	361 (5.9)	345 (6.0)	16 (5.6)	0.898^a^	16 (7.8)	55 (5.9)	0.338^a^
Psychiatric disorders	614 (10.1)	571 (9.9)	43 (15.1)	0.006*^a^	16 (7.8)	110 (11.8)	0.110^a^
Sleep apnoea	500 (8.2)	472 (8.2)	28 (9.8)	0.320^a^	13 (6.3)	79 (8.5)	0.396^a^

*COPD* chronic obstructive pulmonary disease, *FEV*_1_ forced expiratory volume in 1 s, *SD* standard deviation, *CAT* COPD Assessment Test

^*^Statistically significant difference between subgroups

^‡^Random spirometry, assessed without requirement for washout of COPD medication or additional inhalation of short-acting bronchodilator

^†^Exacerbations extrapolated from 6 to 12 months. Comparisons between subgroups performed using ^a^Fisher’s exact test or ^b^Wilcoxon signed rank test

### Outcomes

#### Overall Analysis

Overall, during the 2-year follow-up period only 1697 (28%) patients exacerbated, and many who did exacerbate only had a single exacerbation (977 patients). There was an overall clinically relevant improvement from baseline in mean CAT total score at the end of 2 years (change from baseline − 2.7 [SD 6.4]), with 3529 (58%) patients having a clinically relevant improvement.

There was a significant correlation between the occurrence of exacerbations over 2 years and the change from baseline in CAT total score at Year 2 (*p* = 0.0041). However, the Spearman’s correlation coefficient was only 0.03711, indicating that this correlation was very weak—potentially driven by the high proportion of patients who did not exacerbate. After excluding patients who did not exacerbate during the follow-up period, the correlation was only slightly stronger (0.05952; *p* = 0.0174), perhaps due to the high proportion of patients with only one exacerbation during follow-up.

We therefore decided to examine the relationship in the extremes of the population—patients with frequent or severe exacerbations, and patients with a progressive clinically relevant improvement or worsening in CAT total score.

#### Impact of Frequent or Severe Exacerbations on CAT Total Score

The subgroup of patients with frequent or severe exacerbations comprised only 5% of the overall population (285/6075, Fig. 1). These patients were more likely to be ex-smokers than the remaining patients, to have worse overall lung function and health status at baseline, and a greater occurrence of exacerbations in the six months prior to entry—although 58% didn’t exacerbate during the baseline period (Table 1). In addition, patients in the frequent or severe exacerbations subgroup were more likely to have comorbid bronchiectasis and psychiatric disorders (perhaps as expected); there were no significant differences for any other comorbidities.

**Fig. 2:**
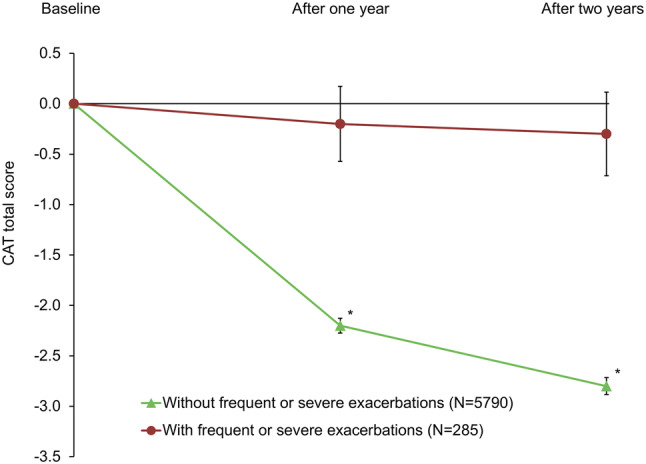
Change from baseline in CAT total score. *p < 0.001. Data are mean change from baseline and standard error of the mean. *CAT* COPD Assessment Test; *COPD* chronic obstructive pulmonary disease

**Fig. 3:**
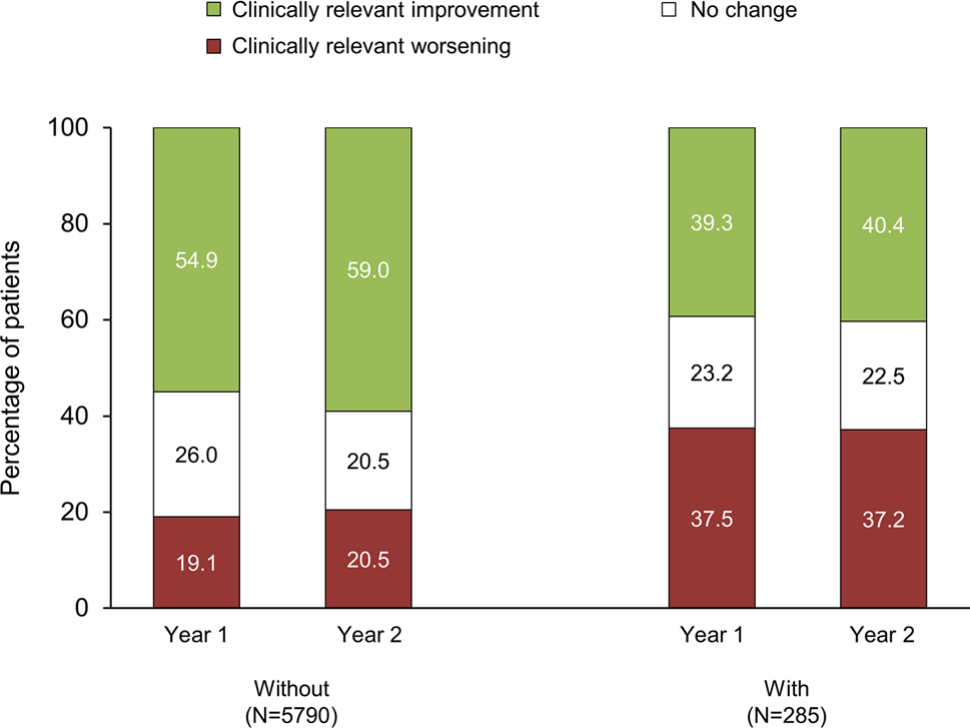
Percentage of patients with a clinically relevant improvement or worsening in CAT total score in the subgroups without or with frequent/severe exacerbations. A clinically relevant improvement is a decrease from baseline of ≥ 2 points; a worsening is an increase from baseline of ≥ 2 points. *CAT* COPD Assessment Test; *COPD* chronic obstructive pulmonary disease

Over the 2-year follow-up period, the subgroup of patients who experienced frequent or severe exacerbations had no overall change from baseline in CAT total score (Fig. 2). In contrast, the remaining patients had overall clinically relevant improvements from baseline at both timepoints, with the differences between the two subgroups being significant at both timepoints (*p* < 0.001 at both). Consistent with the mean data, patients who experienced frequent or severe exacerbations were more likely to experience clinically relevant worsening in CAT total score than the remaining patients (*p* < 0.001; Fig. 3). Furthermore, in the logistic regression analysis the probability of experiencing frequent or severe exacerbations was higher in patients with worsening in CAT total score both in Year 1, and in Year 2, with odds ratios of 1.115 (95% CI 1.09, 1.14; *p* < 0.001) and 1.061 (1.03, 1.09; *p* < 0.001), respectively.

#### Impact of CAT Progression on Occurrence of Exacerbations

Although the majority of patients had an improvement in their CAT total score over the duration of the study, a subset of 205 patients had a progressive worsening (defined as an increase of ≥ 2 units from baseline to the end of Year 1, and a subsequent further increase of ≥ 2 units from the end of Year 1 to the end of Year 2). At inclusion, patients in this progressive worsening subgroup had overall better CAT total scores than those in the progressive improvement subgroup, and were less likely to have an exacerbation history (Table 1). There were no differences between the two subgroups in terms of any comorbidity. The mean (SD) change from baseline in CAT total score after 2 years was + 9.8 (4.3) in the progressive worsening subgroup, compared with − 9.9 (5.0) in the progressive improvement subgroup.

**Fig. 4:**
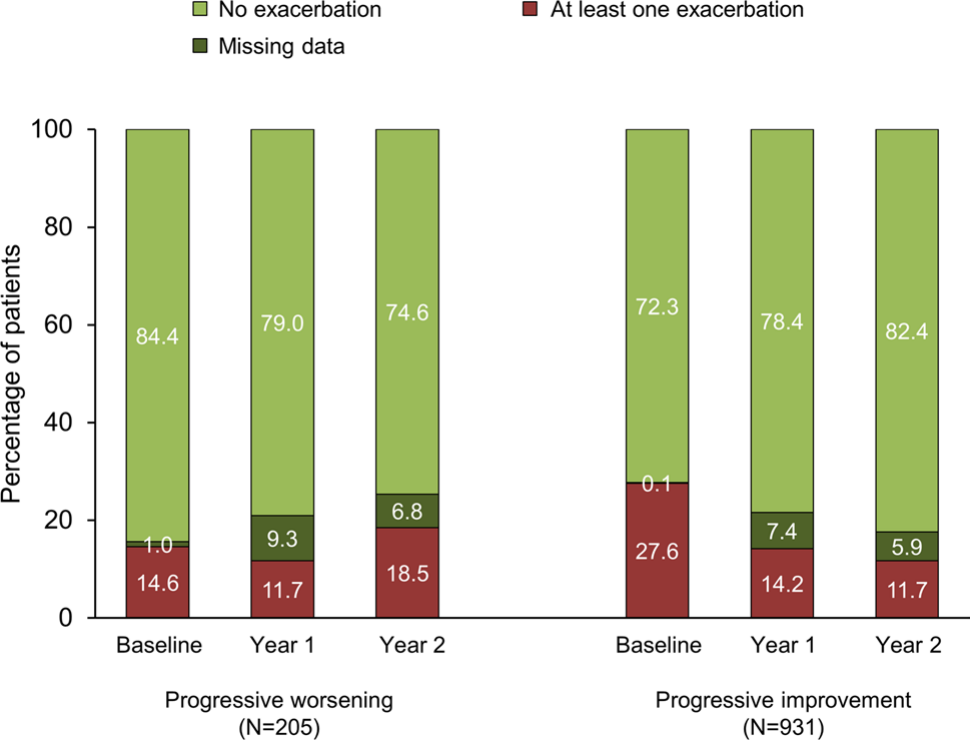
Percentage of patients with exacerbations in the subgroups with progressive worsening or progressive improvement in CAT total score. *CAT* COPD Assessment Test; *COPD* chronic obstructive pulmonary disease

More than three quarters of patients did not exacerbate during the 2-year follow-up (Fig. 4). Patients in the progressive worsening subgroup were more likely to exacerbate during Year 2 than those in the progressive improvement subgroup (18.5 vs 11.7%). However, in the logistic regression analysis, the probability of exacerbating in either Year 1 or Year 2 did not correlate with being in the progressive improvement or worsening CAT subgroups, with odds ratios of 1.015 (95% CI 0.54, 1.90; *p* = 0.962) and 0.596 (0.33, 1.08; *p* = 0.089) in Years 1 and 2, respectively.

## Discussion

Although the short-term impact of exacerbations on CAT total score has been demonstrated in a number of studies, as has the predictive ability of CAT for the occurrence of subsequent exacerbations, to our knowledge this is the first analysis to evaluate the relationship between change in CAT and the occurrence of exacerbations over a 2-year period. Overall, there was a significant correlation between the occurrence of exacerbations and the long-term change from baseline in CAT total score. However, the high proportion of patients with no or one exacerbation meant that this correlation was weak.

We therefore examined the relationship in population extremes. In patients with severe or frequent exacerbations, we were somewhat surprised that overall CAT total score remained unchanged over the course of the 2-year follow-up, with patients equally likely to have a clinically relevant improvement and a clinically relevant worsening. However, in the patients who did not experience frequent or severe exacerbations there was an overall clinically relevant improvement in CAT at the end of the 2-year follow-up, with more than half of the patients having a clinically relevant improvement, and a significant relationship between worsening in CAT over 2 years and an increased risk of frequent or severe exacerbations in the logistic regression analysis. This suggests, therefore, that frequent or severe exacerbations do impact health status—but that the characteristics of the patient population influence the direction of the trajectories. In the current study, one hypothesis could be that by recruiting patients following initiation or change in COPD maintenance medication, overall health status improved, but frequent or severe exacerbations prevented this improvement. In addition, baseline FEV_1_ may have influenced the overall results, given that patients in the frequent/severe exacerbations subgroup were more likely to have worse lung function (FEV_1_ percent predicted). However, as this was a purely observational study, spirometry was conducted according to standard clinical practice—and so these values are not necessarily from post-bronchodilator assessments. Finally, baseline CAT was worse in this subgroup of patients. This is consistent with a previous analysis, in which baseline CAT was higher in patients who subsequently had frequent exacerbations [3]. This difference between the two subgroups complicates interpretation of our data to some extent, since we don’t know whether a 2-unit improvement in CAT from a high (poor) starting value has the same implications for a patient as a 2-unit improvement from a low starting value. In others words, we do not know whether CAT is a linear scale in this population.

Despite the overall health status improvement, a subgroup of patients experienced a progressive worsening in health status, with a clinically relevant worsening from baseline at the end of Year 1, and then a further clinically relevant worsening between the end of Year 1 and the end of Year 2. Compared with the subgroup with a progressive improvement, one notable difference in progressive worseners was exacerbations during the six months prior to entry: more than a quarter of patients in the progressive improvement subgroup exacerbated prior to entry, compared with 15% in the worsening subgroup. During the first year of follow-up, the two subgroups had a similar (and low) incidence of exacerbations, whereas during Year 2 the progressive worsening subgroup was more likely to exacerbate. This suggests that only extreme changes in health status (by definition, at least twice the minimum clinically important difference from baseline after 2 years) are associated with an increased exacerbation risk. Given there were no significant relationships in the logistic regression analysis, this could suggest that exacerbations impact health status, rather than health status impacting exacerbation occurrence. However, as with the frequent/severe exacerbations analysis, the baseline characteristics of the patients complicate interpretation of the data, especially the low proportion of patients with a history of exacerbations, and the differences in baseline CAT total scores. In addition, a ‘ceiling’ or ‘basement’ effect may have influenced the overall results, with a patient who experiences a progressive improvement in CAT total score more likely to start from a high score, whereas a progressive worsener is more likely to start from a low score. It is possible, therefore, that one explanation for our results is that following treatment initiation or change on entry to DACCORD, patients in the progressive improvement subgroup had more opportunity for improvement in their health status.

Although the ‘real life’ and purely observational nature of DACCORD is a strength, such a design does have limitations. Most importantly, the only data collected are from standard clinic visits, and it is not possible to mandate assessments or introduce additional parameters. For example, DACCORD is not able to require washout of COPD medication or post-bronchodilator data. Of course, these are the data that clinicians use to make prescribing decisions. Secondly, to recruit as broad a population as possible, minimal inclusion and exclusion criteria were applied. Although this means that the population we recruited was representative of a ‘real life’ population, we were not able to actively recruit patients with an exacerbation history (who would be at increased risk of future exacerbations [12]). In the event, the population recruited into DACCORD had a high proportion of patients who did not exacerbate at all, which makes examination of the correlation between exacerbations and change in health status challenging. Importantly, however, a similar (low) occurrence of exacerbations has been recorded in other ‘real life’ populations, such as SPIROMICS [13]. Furthermore, patients were recruited into DACCORD following change or initiation in medication, which might explain why there was an overall improvement in health status—again, this makes interpretation of the results challenging, and is one reason why we decided to analyse the two progressive CAT subgroups.

## Conclusion

At least in the DACCORD population (newly initiating or changing maintenance COPD medication), patients with frequent or severe exacerbations experienced a long-term worsening in health status (beyond the acute effect of an exacerbation) compared with patients who do not exacerbate—although this difference was mainly due to an improvement in the non-exacerbating group. This suggests that patients with frequent or severe COPD exacerbations are a distinct phenotype. However, following initiation or change in COPD maintenance medication, exacerbations are rare events and single exacerbations do not appear to have a prolonged effect on health status.

## Abbreviations

CAT: COPD Assessment Test
COPD: Chronic obstructive pulmonary disease
DMP: Disease Management Program
FEV_1_: Forced expiratory volume in 1 s
SD: Standard deviation
